# Supplementary material for: Complexome profiling of the Chlamydomonas psb28 mutant reveals TEF5 as an early PSII assembly factor
Source: Plant Cell. 2025 Mar 17;37(6):koaf055. doi: 10.1093/plcell/koaf055 (PMC12164590; doi:10.1093/plcell/koaf055)
Supplement: koaf055_Supplementary_Data [file koaf055_supplementary_data.zip › psb28_interactive.zip!/plots/intensity/qualityPlots/peptides/psbH_Cre-1.g2717030.t1.p1.html]

### Cre-1.g2717030.t1.p1

Trivial name: psbH  
SeqCoverage [median]: 36.40  
Mass: 9.44 kDa  
  
 

| Sequence | MissedCleavage | Mass [Da] | wtIntensity | muIntensity | Score |
| --- | --- | --- | --- | --- | --- |
| AKPSKVNSDFQEPGLVTPLGTLLRPLNSEAGK | 3 | 3362.82 | 1513.60 | 0.00 | 67.28 |
| PLNSEAGK | 0 | 814.42 | 16716.70 | 1579.37 | 122.19 |
| VNSDFQEPGLVTPLGTLLR | 0 | 2055.10 | 16089.04 | 0.00 | 150.11 |
| VNSDFQEPGLVTPLGTLLRPLNSEAGK | 1 | 2851.51 | 297044.33 | 0.00 | 147.17 |
|  |  |  | 331363.7 | 1579.4 |  |
